# The impact of initial cooling rates on cell preservation in frozen water-dimethyl sulfoxide media: a morphological study

**DOI:** 10.1007/s44211-025-00815-8

**Published:** 2025-07-04

**Authors:** Rinko Sabanai, Yoshifumi Suzuki, Takafumi Mizushige, Nobuo Uehara, Arinori Inagawa

**Affiliations:** 1 School of Engineering, Utsunomiya University, 7-1-2, Yoto, Utsunomiya, Tochigi 321-8585 Japan; 2 School of Agriculture, Utsunomiya University, 350, Minemachi, Utsunomiya, Tochigi 321-8585 Japan

**Keywords:** Cryopreservation, Freeze-concentrated solutions, Cell viability, Morphological study

## Abstract

We investigated the morphological features of a freeze-concentrated solution (FCS) formed in frozen aqueous dimethyl sulfoxide (DMSO) solutions for cell cryopreservation. Specifically, we investigated the effects of cooling rates and initial DMSO concentrations on FCS morphology. At a cooling rate of 1 ℃/min, relatively large FCS channels formed owing to the crystallization of the extracellular ice crystals, accommodating cells effectively. Conversely, rapid cooling rates resulted in fine ice crystals, leading to the formation of narrower FCS channels. Slow cooling promoted cell accommodation, attributed to ice crystal reorientation and larger FCS sizes, underscoring the impact of FCS width profiles on cell accumulation during freezing. The recovery of C2C12 myoblasts was also studied by altering the freezing rate. The cell recovery rate of C2C12 myoblasts demonstrated that slow cooling improved recovery, whereas rapid cooling decreased it. Medium cooling rates showed greater variability in recovery, suggesting reduced consistency in cryopreservation outcomes. While numerous factors determine cell viability during freezing processes, our findings highlight FCS morphology as a critical determinant in designing effective cryopreservation protocols.

**Graphical abstract:**

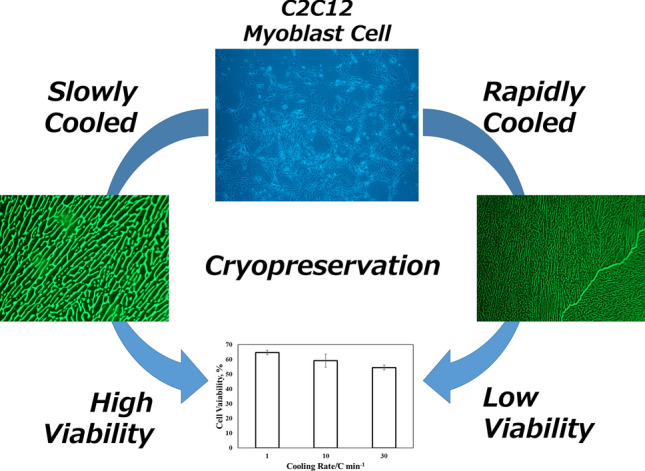

**Supplementary Information:**

The online version contains supplementary material available at 10.1007/s44211-025-00815-8.

## Introduction

Preserving biomolecules, including DNA, proteins, and cells, is of great interest to biologists and physicians because external environmental factors easily degrade these molecules [1, 2]. Recent progress in regenerative medicine has increased the demands for long-term preservation of stem cells and tissues, exemplified by the establishment of cell banking [3, 4]. Numerous preservation methods for biological cells and tissues have been proposed, including in situ organ preservation [5], high-subzero preservation [6], and programmed metabolic suppression [7]. Cryopreservation has been widely employed for long-term preservation [8]. This technique generally involves the addition of a cryoprotectant to the sample and carefully programming the cooling process. Compared to other preservation techniques, cryopreservation can extend the preservation period to a decade [9]. However, the freezing process is inherently stochastic, indicating that ice nucleation is hardly controllable in a bulky solution sample. To address this, numerous studies have focused on optimizing freezing protocols to achieve high cell viability, considering parameters such as cooling rate and preservation containers [10–12].

Among the numerous parameters considered for optimization, two are most frequently examined: the cryoprotectant and cooling rate. Cryoprotectants range from small molecules to macromolecules. A representative small molecule cryoprotectant is dimethyl sulfoxide (DMSO) [13, 14]. DMSO permeates the cell through membranes and inhibits intracellular ice nucleation [15]. Macromolecular cryoprotectants, including hydroxyethyl starch [16], serum proteins [17], dextran [18, 19] and polyethylene glycol [20] are also commonly used. Compared to DMSO, these materials are less toxic to cells, leading to high cell viability. Naturally occurring antifreeze proteins are also one of the applications of cryoprotectants [21, 22]. These proteins inhibit ice nucleation and suppress ice recrystallization during the thawing process through Ostwald ripening [23]. Cooling rates are another important factor in cryopreservation. Generally, the cooling process for cryopreservation consists of two steps: slow freezing and immersion of samples in liquid nitrogen. It has been reported that cell recovery and reproducibility are relatively high for a slow cooling rate. [24]

The cryopreservation process involves freezing the solution medium, during which the morphology of ice crystals on freezing aqueous solutions is strongly influenced by the solute concentration and freezing speed, which are exactly the key parameters mentioned earlier. When an aqueous solution freezes, phase separation occurs, generating pure ice crystals and a liquid phase where solutes become concentrated [25]. The liquid phase is called the freeze-concentrated solution (FCS). The initial solute concentration and freezing speed strongly influence the size and shape of the FCS [26]. Our research group has been studying the morphology of the FCS and attempting its use in separation fields for biomaterials and investigation of the ice/solution properties [27–33]. In particular, we have successfully introduced nano- and microparticles into the FCS by freezing dispersions and controlling the growth of ice crystals through external factors such as freezing temperature and seeding for ice nucleation [27, 33]. When the freezing speed is too fast, the FCS adopts a small and pore-like morphology [26, 34, 35]. This condition reduces the probability of cell accumulation as the FCS volume becomes insufficient. If cell accumulation is insufficient, the effect of cryoprotectants on the cells is inhibited, which may affect the cell recovery rate. Therefore, understanding the correlation between cell viability during cryopreservation and the morphological features of the FCS, where cells accumulate during the freezing process, is essential for optimizing cryopreservation protocols.

To understand the effect of FCS morphology on cell viability during cryopreservation, we conducted microscopic studies of the FCS formed in frozen DMSO solutions under various conditions. The cooling rate and initial concentration of DMSO were examined as factors shaping the morphological features. We focused on the change in the FCS morphology during the freezing process by continuous monitoring. The width of the FCS was statistically analyzed, and its profile was discussed. Furthermore, we evaluated the accumulation behavior of cells subjected to different freezing conditions and analyzed the recovery of mouse myoblast cells during cryopreservation to determine the correlation between morphological features and cell viability.

## Experimental section

### Chemicals

Dimethyl sulfoxide (DMSO) (Kanto Chemical Co., Inc. Tokyo, Japan), sodium fluorescein (FUJIFILM Wako Pure Chemical Corporation Osaka, Japan), a cell viability assay kit (Cell Counting Kit-8; Dojindo Laboratories Japan) were used. All aqueous solutions were prepared using ultrapure water obtained using a Milli-Q system (Merck, USA). Yeast cells were obtained from Nisshin Seifun Welfare, Inc. (Japan).

### Microscopic observation of the FCS

**Fig. 1:**
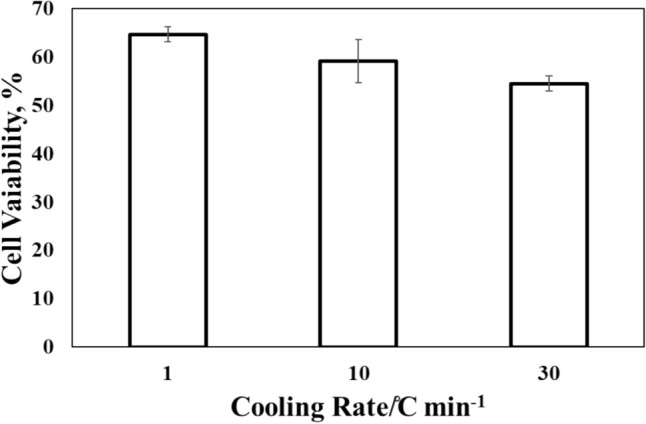
The cell viability as a function of cooling rate during freezing

The microscopic setup is shown in Fig. 1. An upright fluorescent microscope (BH-2, Olympus, Japan) equipped with a CMOS camera (AdvanCam-E3Rs, Advan Vision, Japan) and a cooling stage (10,002 L, Japan Hightech Co., Ltd., Japan) was used. As an objective, a 4 × objective lens (DPlan 4, Olympus, Japan) was used. A 10 μL aliquot of a 100 μM sodium fluorescein solution in DMSO was sandwiched between two slide glasses (Matsunami Glass Ind., Ltd., Japan) and placed on the cooling stage. The solution was cooled to an appropriate speed, which was controlled by a computer. Morphological observations were made using fluorescence microscopy. To examine cell accumulation during freezing, rabbit red blood cells (Cosmobio, Japan) dispersed in DMSO were frozen using the same cooling procedure. The concentration of DMSO was set to 5, 10 and 20 wt%. In general, the condition with 5 wt% is frequently used whereas the upper limit of the concentration is considered to be 10 wt% due to the toxicity of the DMSO [36]. Therefore, we investigated the conditions frequently employed. Transmission microscopic images were acquired by irradiation with blue excitation light to visualize the FCS stained with sodium fluorescein.

### Evaluation of the FCS channel width and ice particle size

The width of the FCS was measured with ImageJ software with the fluorescence images. The 40 targeted FCS channels were selected not to overlap for counting manually. The histogram of the channel width was analyzed by the Gaussian function. The size of the ice particle was analyzed by the particle analyzing method pre-installed in ImageJ software.

### Cell cultivation

C2C12 myoblasts (RIKEN BioResource Center, Ibaraki, Japan) were cultured at 37 ℃ under a 5% CO_2_ atmosphere in growth medium consisting of Dulbecco’s Modified Eagle’s Medium (DMEM; FUJIFILM Wako Chemicals) supplemented with 10% fetal bovine serum (Thermo Fisher Scientific), and 1% penicillin–streptomycin (FUJIFILM Wako Chemicals). A transparent image of the cell is shown in Fig. S2.

### Measurement of cell viability

To evaluate cell viability using a trypan blue staining assay. A 1:1 mixture of 4 × 10^5^ cells/mL of cells and 0.4% trypan blue solution (FUJIFILM Wako Chemicals) was prepared. The number of viable or dead cells was counted using a cell counting chamber (Hirschmann, Germany), and cell viability was calculated as the ratio of viable cells to total cells.

## Results and discussion

### Cell recovery and its relationship between the morphological study

First of all, we examined the effect of the cooling rate on the cell recovery. C2C12 mouse myoblasts were used as model cells. Generally, cell preservation by freezing is initiated by slow cooling, followed by immersion in liquid nitrogen for long-term preservation. When cells are used, a fast-thawing process is required to minimize the physical damage caused by ice crystals. In this study, the freezing–thawing process was replicated using a temperature-controlled stage. Fig. S3 shows a schematic representation of the temperature operation of the cell viability assay. Briefly, the DMSO solution dispersing the cell was freezing at a given cooling rate until the temperature reached −60.0 ℃. The temperature was decreased to −100 ℃ at a cooling rate of 50 ℃/min to imitate fast freezing in the liquid nitrogen. The temperatures were kept for 2 min, and the frozen samples were thawed at the speed of 50 ℃/min to minimize the effect of ice nucleation and related cellular damage. The cells were stained with trypan blue, and the ratio of viable cells to total cells was determined.

Figure 1 shows a comparison of the cell recovery at different cooling rates. When the cooling rate was set to 1 ℃/min, the cell viability was 65%. On the other hand, a fast-cooling rate of 10 and 30 ℃/min reduced the viability to 59% and 54%. The results were analyzed using analysis of variance (ANOVA). The obtained P value was 0.034, which was lower than 0.05 at the significant level of 5%. The ANOVA results confirmed a significant difference between each condition. Since the fast-freezing process after reaching eutectic points and the subsequent thawing process are unified in all conditions, the difference in viability should be attributed to the initial freezing processes.

### Morphological study of the FCS formed in frozen DMSO-water binary media

The morphological features of FCS at the cooling rate under optimized solution conditions for cell preservation were studied by fluorescence microscopy. First, we observed morphological changes in the FCS concerning the cooling rate. While slow cooling (ca. 1 ℃/min) is generally preferred for cells cryogenically preserved in frozen media. [36–38], the impact of cooling rate on FCS size and morphology is less well understood. Although frozen media seem to reach an equilibrium state after the media are frozen macroscopically, the microscopic structure of ice crystals continues to grow to reduce the interfacial energy. Therefore, the morphology of the FCS is determined by the balance between this reduction in surface free energy and the thermodynamic equilibrium of the system.

**Fig. 2:**
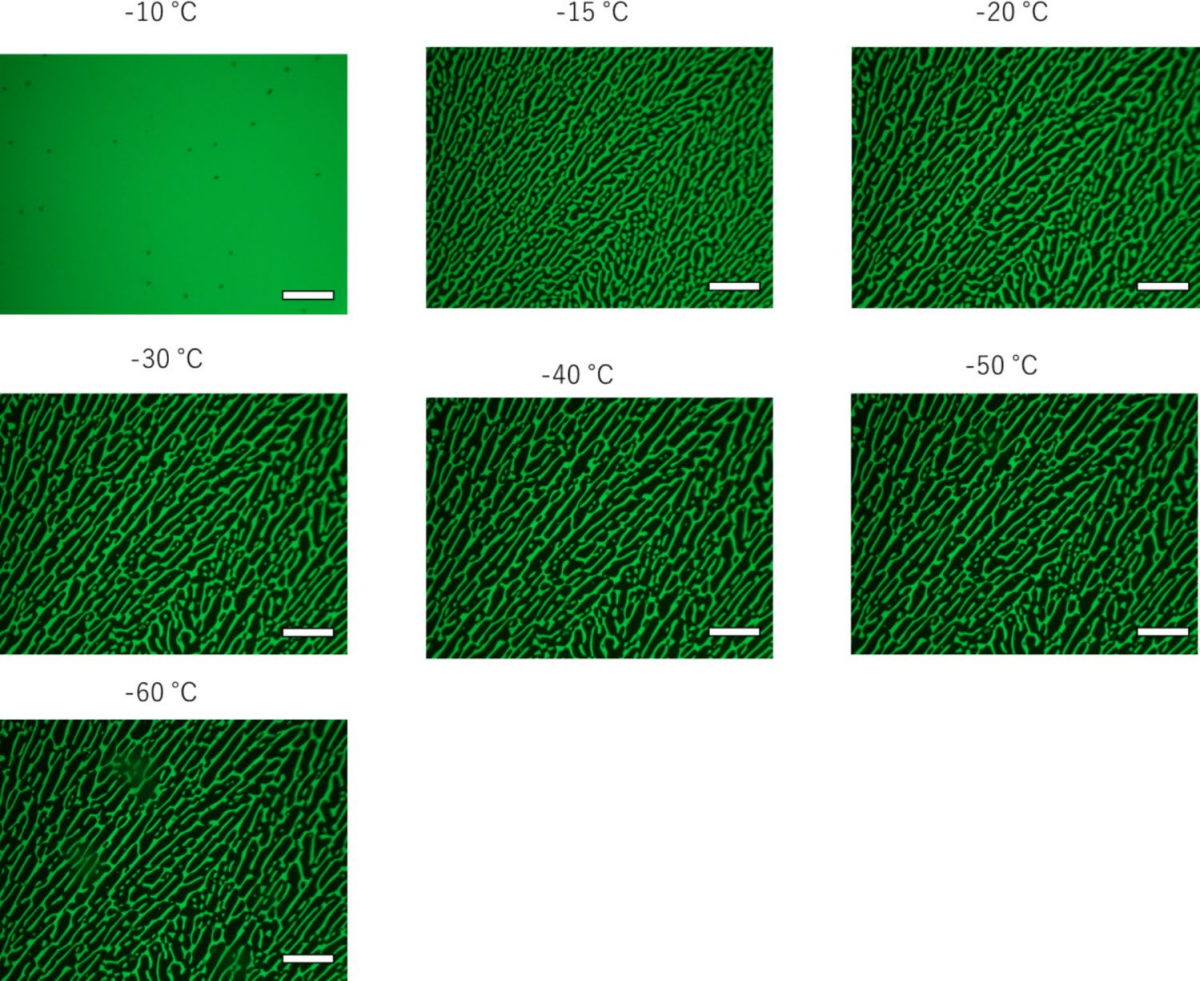
Fluorescence microscopic images of the FCS frozen at 1.0 ℃/min at every 10 ℃ during freezing. 10 wt.% aqueous DMSO solution containing 2 mM sodium fluorescein. Scale bar: 100 μm

Herein, we examined three different cooling rates (1.0, 10.0, and 30.0 ℃/min). We compared the morphological features of the FCS at a given temperature. Figure 2 shows the fluorescence microscopic images of the FCS frozen at 1.0 ℃/min captured at every 10 ℃ interval. Sodium fluorescein, excluded from the growing ice crystals, concentrates within the FCS, allowing its visualization in green. By observation with the naked eye, freezing occurred at ca. −11 ℃ for each freezing process. Note that the freezing phenomena occurs after the solution had reached the supercooling conditions. After freezing, numerous fine ice crystals appeared at −15 ℃, which then merged into larger crystals as the temperature decreased. Simultaneously, the width of the FCS surrounding the ice crystals increased. This process is attributed to the reduction in solid–liquid interfacial energy. Once the FCS morphology stabilized, the FCS width gradually decreased until the temperature reached −60 ℃. This reduction is due to the thermodynamic equilibrium of the FCS [27]. The degree of reduction can be determined using the phase diagram of the water-DMSO binary system. [38, 39]

Next, we examined the effect of the cooling rate on the morphological features of the FCS. Figures S4 and S5 show the representative fluorescence microscopic images of the FCS frozen at 10.0 and 30.0 ℃/min, respectively. Note the brighter lines shown in Fig. S5 is due to the FCS spontaneously poured into the space between polycrystallines. Interestingly, the growth of the ice crystals was less prominent compared to the cooling rate of 1.0 ℃/min. This difference is attributed to the short time required for Ostwald ripening of ice particles to maximize the particle sizes. A comparison of the morphological features suggests that the balance between the reduction of the surface area by Ostwald ripening and FCS volume determined by the FCS concentration determined by two-phase diagram, which can be externally controlled by the cooling rate.

**Fig. 3:**
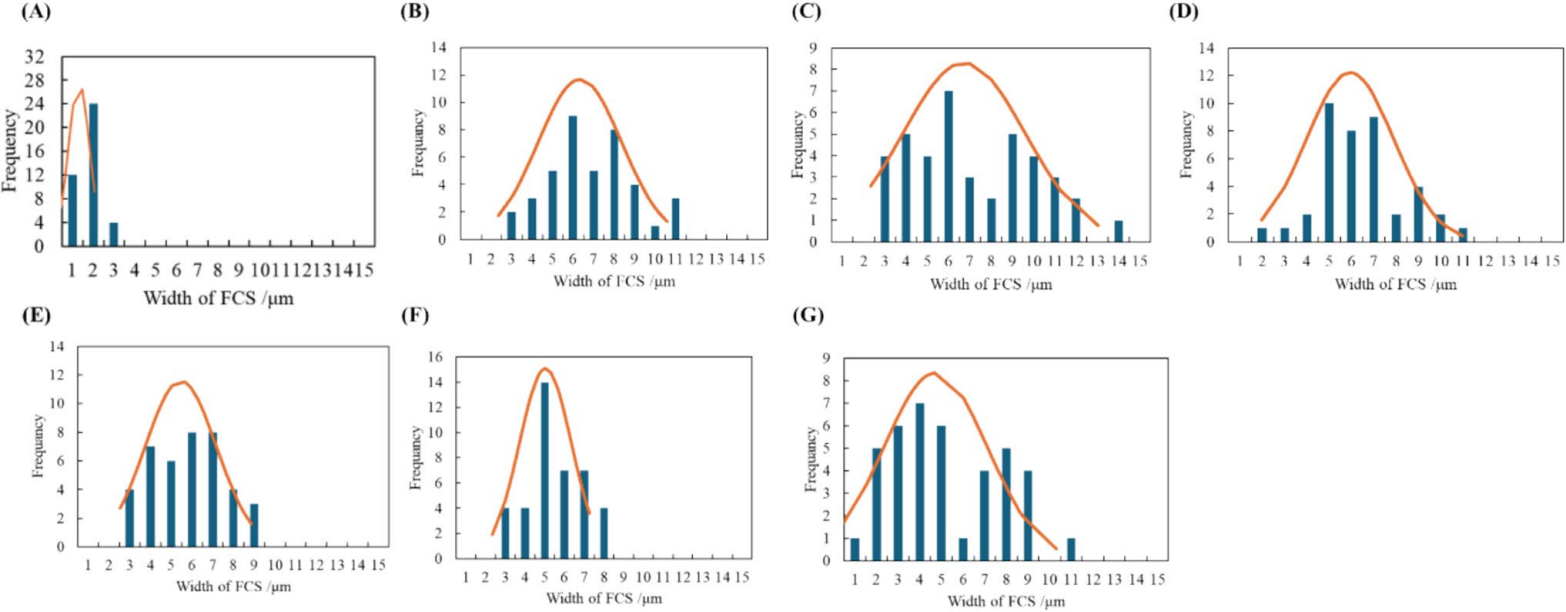
Histogram of the FCS width measured from the microscopic images at different 200 points at every 10 ℃ on freezing processes when frozen at 1.0 ℃/min. **A** −11℃ **B** −15 ℃ **C** −20 ℃ **D** −30 ℃ **E** −40 ℃ **F** −50 ℃ **G** −60 ℃

**Fig. 4:**
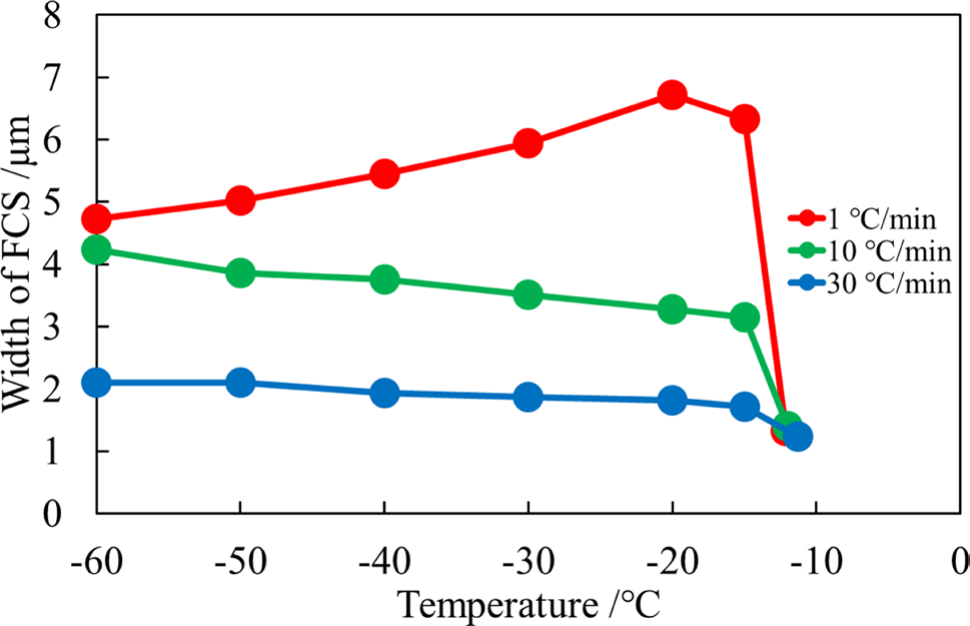
The change in the FCS width at every observation temperature

The balance between these two processes can be assessed by measuring the size of the FCS. Herein, we statistically evaluated the change in FCS width during freezing process statistically. The FCS width was measured using ImageJ software at 40 points on microscopic images captured under identical cooling conditions. Each experiment was repeated five times at a constant cooling rate. The measured width was analyzed using Gaussian functions to obtain average widths and variances. Figure 3 shows the histogram of the FCS width measured from the microscopic images at every 10 ℃ during freezing at 1.0 ℃/min. Due to experimental deviation caused by experimental errors, the position of crystallization and freezing speed, the histogram might not be fully expressed with the Gaussian functions. However, the total volume of the FCS in the system is definitively determined based on the phase diagram. Therefore, we herein assumed that the histogram can be fitted with a single Gaussian function. The identical average and central width values indicate that the FCS width distribution is well-explained by Gaussian functions. Similarly, the FCS widths for cooling rates of 10.0 and 30.0 ℃/min are summarized in Fig. S6. The average FCS widths, as a function of temperature, are shown in Fig. 4. The change in width at the measured temperature showed different profiles. At a slow cooling rate of 1.0 ℃/min, the initial FCS width was 6.3 μm, increasing as the temperature decreased from −10 to −20 ℃. The FCS width reached −20 ℃ before gradually reducing to 4.7 μm at −60 ℃. Conversely, at a faster cooling rate of 10.0 and 30.0 ℃/min, the initial widths were 3.1 and 1.7 μm, respectively, with only slight increases during cooling. The width showed a slight increase until the temperature reached −60 ℃ continuously. The final width was ca. 4.2 and 2.1 μm, respectively, which depended on the cooling rate.

Generally, the width of the FCS is determined by the initial concentration of the solutes and the operation temperature. According to the phase diagram of the binary solution system, the volume ratio of the FCS to ice can be expressed as the following equation.

1 $$\frac{{V}_{\text{FCS}}}{{V}_{\text{ice}}}=\frac{{\rho }_{\text{ice}}}{{\rho }_{\text{FCS}}}\frac{{C}_{\text{ini}}}{{C}_{\text{FCS}}-{C}_{\text{ini}}}$$

where *V*_ice_, *V*_FCS_, *c*_ini_, *c*_FCS_, *ρ*_ice_ and *ρ*_ice_ are the volume of ice and the FCS, the initial and FCS concentrations of DMSO, and the volumetric mass density of FCS and ice, respectively. This phase diagram is interpreted by the Gibbs free energy of the solute dissolving. [40] Thus, if the system temperature is quasi-statically decreased, the FCS volume is reduced. Assuming the morphology of the FCS, i.e., ice crystals, is not changed, the FCS width is subsequently reduced. [27] However, our results show different profile from this assumption. Thus, the change in the.

**Fig. 5:**
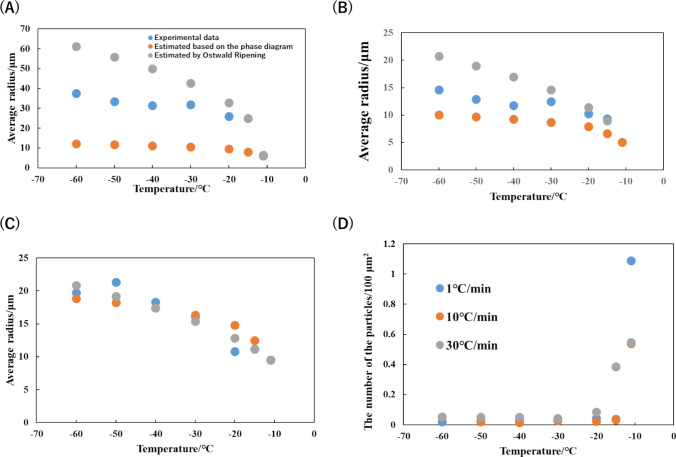
The radius and the number of the ice particles. Average radius of the ice particles under the condition of **A** 1℃/min, **B** 10℃/min, **C** 30℃/min. **D** The number of the ice particles

Herein, we interpreted this profile by Ostwald ripening of ice crystals. Generally, the change of particle size induced by Ostwald ripening can be predicted by the following equation.

2 $${\langle R\rangle }^{3}{-\langle {R}_{0}\rangle }^{3}=\frac{8\gamma {c}_{\infty }{v}^{2}D}{9{R}_{G}T}t$$

where < *R* > , < *R*_0_ > , γ, *c*_∞_, v, D, *R*_g_, T, and t are the average radius of ice particles, the average radius of ice particles at initial condition, interfacial energy of ice/water interface (0.016 J/m^2^), the molecular volume, the diffusion coefficient, the gas constant, the absolute temperature and the time. We measured the size of the ice particles. To simplify the analysis, we calculated the virtual radius of ice particle by assuming that ice particle has spherical shape. Figure 5(A–C) shows the average radius of the ice particles generated by freezing 5 wt% DMSO solution at various cooling rates. The estimated profiles of the radius both based on the phase diagram (with Eq. (1)) and the Ostwald ripening theory (with Eq. (2)) are shown as well. Since Ostwald ripening reduces the number of the particle, we also counted the number of the ice crystals at each temperature, which is shown in Fig. 5D. As for the results with 1℃/min and 10℃/min, the radius profile is similar to the estimated values based on the Ostwald ripening theory until the temperature reaches −20℃. The number of the ice particle significantly reduced in this temperature regions. The profile then obeys the estimated values based on the phase diagram. In this temperature regions, the number of the ice particles does not change. In the case of 30℃/min, both the estimated radius shows similar value each other. This is because the freezing rate is fast. The time for Ostwald ripening is thus not enough to grow the ice particle larger. The number of the ice particles is also reduced, supporting that Ostwald ripening is still occurred. Therefore, this profile difference indicates that the Ostwald ripening plays an important role to determine the morphology of the ice particle i.e., the FCS in this freezing conditions. When the cooling rate is slow, Ostwald ripening is promoted, inducing the fusion of ice particle the reduction of the number of the crystals. When the cooling rate is high, the degree of the fusion by Ostwald ripening is reduced compared to the slow cooling rate. Yet, the Ostwald ripening is still works. However, fast cooling rate does not provide quasi-static cooling to the frozen system, remaining the larger effect of the Ostwald ripening than that of ice/FCS binary equilibrium.

The protocol for cryopreservation requires an optimized cooling rate before immersing the sample into liquid nitrogen, ideally 1–5 ℃/min [8]. The ideal cooling rate provides a large width of the FCS enough to accumulate the cells, whose diameters are typically several micrometers. Conversely, a narrow FCS reduces the probability of cell accumulation, inhibiting the effect of cryoprotectants on the cell, and physical interaction with extracellular ice crystals has a greater impact on the cells.

**Fig. 6:**
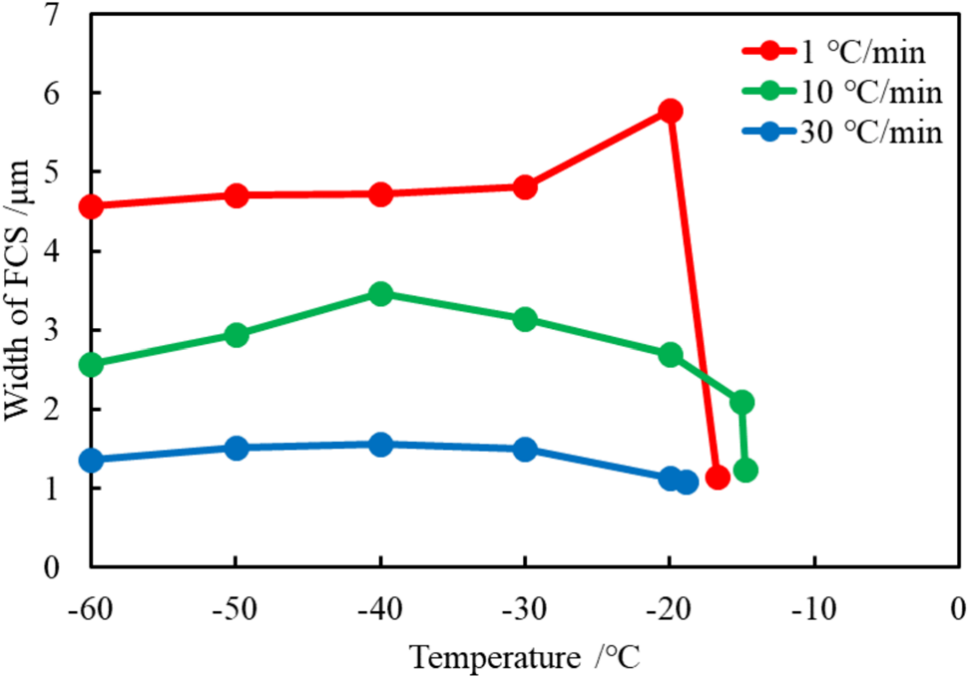
The change profile in the FCS width depends on the initial 20 wt% DMSO concentrations

The FCS width can also be modulated by adjusting the initial concentration of DMSO as explained by Eq. (1). Here, we altered the initial concentration of DMSO to assess the FCS. Figure S7 shows a fluorescence microscopic image of the FCS when the initial concentration of DMSO was set to 20 wt.%. The width of the FCS was statistically evaluated based on a Gaussian distribution, similar to the analysis described above. Figure 6 compares the FCS width change profile depending on the initial DMSO concentration. Based on the phase diagram, freezing temperature is altered compared to the condition with 10 wt%. The FCS width profile is identical to that shown in Fig. 4. Note that the absolute value of the width of the FCS should larger than the values in Fig. 4 according to Eq. 1. However, this assumption makes sense when the number of the ice particles is identical each other. Nevertheless, higher DMSO concentration is reported to be harmful to the cell, which may reduce cell viability [41]. Generally, the upper concentration of DMSO as a cryoprotectant is considered to be 10 wt% [2]. Thus, although higher DMSO concentrations yield larger FCS regions, the concentration should be minimized as much as possible to reduce the probability of a cell being affected by the toxicity of DMSO.

### Cell compartment in the FCS on freezing

**Fig. 7:**
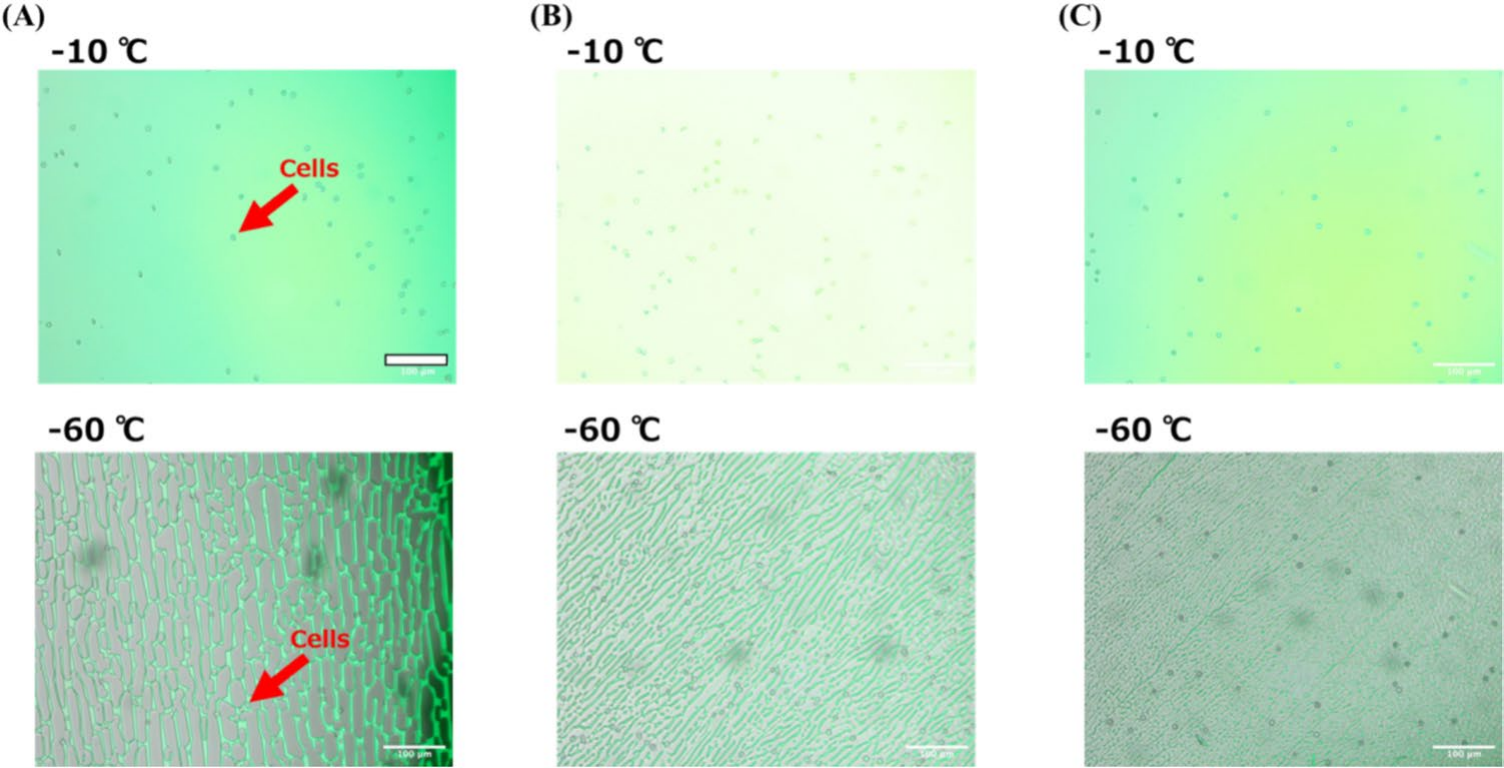
The representative images of yeast cells in a 10 wt.% DMSO solution during freezing showing cell accumulation in the FCS. Freezing speed of **A** 1 ℃/min, **B** 10 ℃/min and **C** 30 ℃/min

The morphology and changes in the FCS may influence cell accumulation. In this study, we examined the behavior of cells in a freezing medium. As a model cell, rabbit red blood cells (*d* = ca. 5 μm) were employed for clear observation. Figure 7 compares microscopic images of the frozen DMSO solution containing blood cells and sodium fluorescein. To visualize the FCS using fluorescein, the sample was irradiated with blue excitation light during the transmission observation. Therefore, the position of the FCS could be easily observed by recognizing the green color in the images. When the cooling rate was set to 1 ℃/min, most cells were accumulated in the FCS, the width of which is larger than the cells even at −60 ℃. However, the cells are excluded from the FCS for the experiments with the cooling rate of 10.0 and 30.0 ℃/min. Even though the initial morphology just after the freezing is similar, as shown in Figs. 2, S4, and S5, the accumulation of the cells is more prominent at the cooling rate of 1.0 ℃/min than in other experimental conditions. The Ostwald ripening of the external ice crystals increased the size of the FCS. This process is obvious when the cooling rate is low because sufficient time is provided for recrystallization, as shown in Fig. 4. The accumulation of cells in the FCS plays an important role in maximizing the efficacy of cryoprotectants. If cells are excluded from the FCS, this protective process is hindered, and external ice crystals exert greater physical damage on the cells. Consequently, cell viability decreases owing to ineffective cryoprotectant function. As discussed above, the cooling rate strongly affects the morphological features of the FCS. For a slow cooling rate, the size of the FCS was relatively large, equivalent to that of the cells. Conversely, fast freezing produces narrow FCS channels smaller than the cell size. The size dependence of the cells determines the efficiency of cell accumulation in the FCS. Proper accumulation leads to the efficient dehydration of cells for successful cryopreservation. Conversely, a small volume of FCS reduces the accumulation probability, suppressing the opportunity for cells to be exposed to the cryoprotectant. The small volume of FCS also induces physical damage to cells during the freezing process. The exclusion process of the cell during freezing showed that the wide FCS could fully accumulate the cells therein. Conversely, the cells were surrounded by ice crystals. Therefore, the above results suggest a correlation between cooling rate and cell viability from the viewpoint of FCS morphology. Moreover, the variance in cell viability for a cooling rate of 10 ℃/min is larger than in other conditions. This could be attributed to the intermediate size of the FCS formed upon freezing, which is comparable to the cell size. This comparative size leads to a stochastic behavior of cell accumulation.

Summarizing the above results, the correlation between the cooling rates during cryopreservation and cell viability can be described as follows: During the FCS formation, the ice crystals were fine, and the FCS size was relatively small during the freezing process at any cooling rate. For a slow cooling rate, Ostwald ripening of the extracellular ice crystals occurred to reduce the ice/solution interface area, increasing the FCS. After the interfacial energy was minimized, the FCS began to reduce in volume to reach thermodynamic equilibrium. Because Ostwald ripening maximizes the FCS size, several cells have room to accumulate. Conversely, a fast-cooling rate reduces the time required for Ostwald ripening, which does not allow the FCS size to be maximized by recrystallization. The FCS was so small that efficient cell accumulation was not observed. This inhibits favorable processes for cryoprotection by cryoprotectants. Medium cooling rates affect cell viability because the FCS size is comparable to the cell volume. The morphology of the FCS and its change during the freezing processes regulated by the cooling rate can be considered an important factor correlating cell viability with the cryopreservation methodology.

## Conclusion

We investigated the morphological characteristics of the FCS formed in DMSO-water binary media under generally optimized conditions for cell cryopreservation. The result demonstrated that FCS morphology and its changes during the freezing process are strongly influenced by the cooling rate and initial concentration of DMSO. At slow cooling rates, wide FCS channels that are capable of accumulating cells were formed, even near the eutectic point. Conversely, fast freezing produces a narrow FCS smaller than that of biological cells. Cell accumulation was observed at slow cooling rates. Cell viability was then assayed using C2C12 myoblast cells frozen at different cooling rates. The slow cooling rate increased cell viability, indicating a positive correlation between the freezing process, FCS size, and cell viability.

In this study, we focused on the morphological features of the FCS, which is an extracellular phenomenon. Numerous factors have significant effects on cryopreservation. These include dehydration, ice nucleation, and Ostwald ripening. Although these processes were not discussed in the present study, studies using NIR or Raman spectroscopy will reveal the hydration state of the cell during the freezing process, which will be reported in future work.

## Supplementary Information

Below is the link to the electronic supplementary material.

Supplementary file1 (DOCX 2799 KB)

## Data Availability

Data are available on reasonable requests.
