# Supplementary material for: The impact of initial cooling rates on cell preservation in frozen water-dimethyl sulfoxide media: a morphological study
Source: Anal Sci. 2025 Jul 4;41(9):1555–63. doi: 10.1007/s44211-025-00815-8 (PMC12408769; doi:10.1007/s44211-025-00815-8)
Supplement: Supplementary file 1 — Supplementary file1 (DOCX 2799 KB) [file 44211_2025_815_MOESM1_ESM.docx]

Supplementary Material

Note

**The impact of initial cooling rates on cell preservation in frozen water-dimethyl sulfoxide media: A morphological study**

Rinko Sabanai^1^, Yoshifumi Suzuki^2^, Takafumi Mizushige^2^, Nobuo Uehara^1^, Arinori Inagawa^1^,*

*1. School of Engineering, Utsunomiya University, 7-1-2, Yoto, Utsunomiya, Tochigi 321-8585, Japan.*

*2. School of Agriculture, Utsunomiya University, 350, Minemachi, Utsunomiya, Tochigi 321-8585, Japan*

_*_Corresponding to: ainagawa@cc.utsunomiya-u.ac.jp (A. Inagawa)


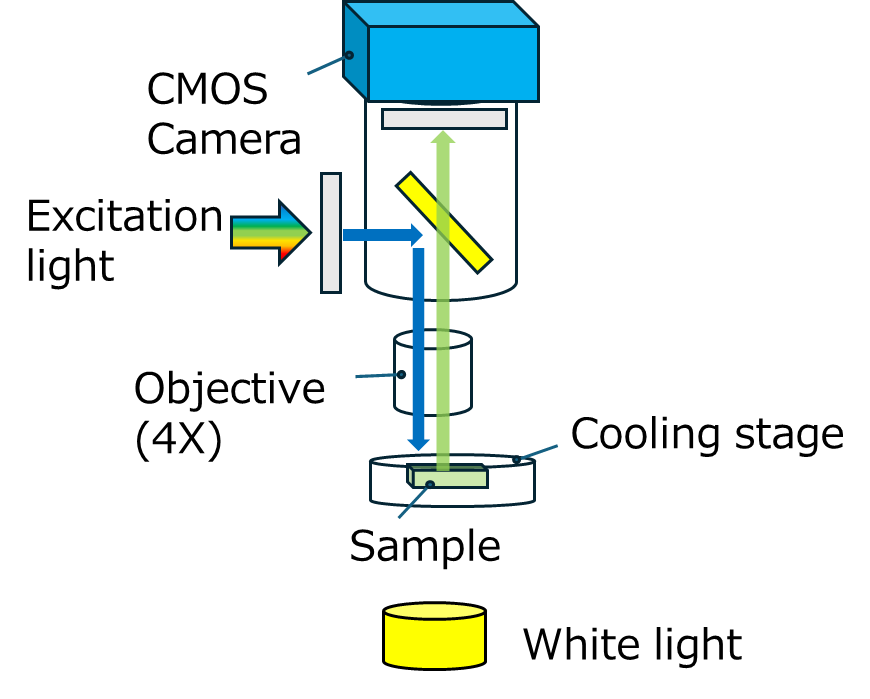


**Figure S1.**　Schematic illustration of the microscopic setup


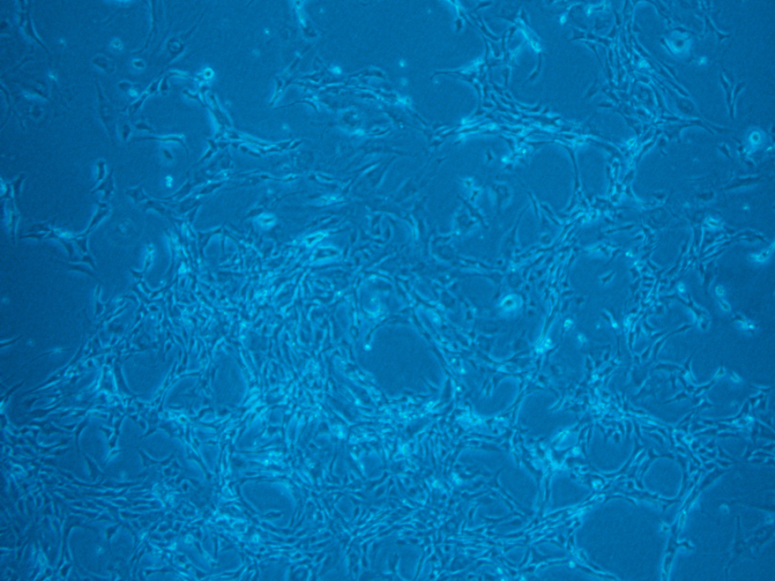


**Figure S2.**　Transmission images of the C2C12 cells used for cell viability assays. The cells are adhered to the petri dish in this condition.


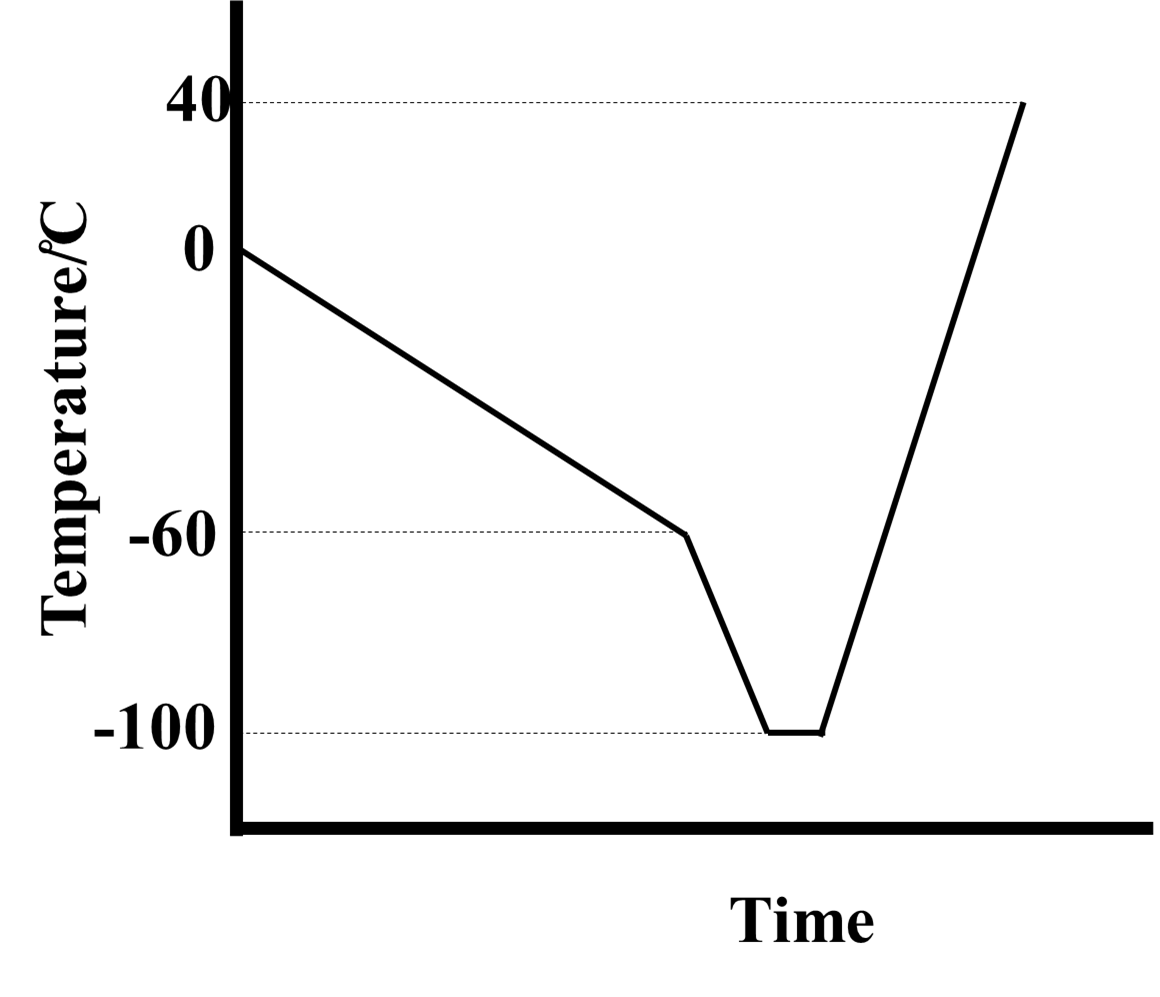


**Figure S3.** The schematic diagram of the temperature operation on cell viability assay.


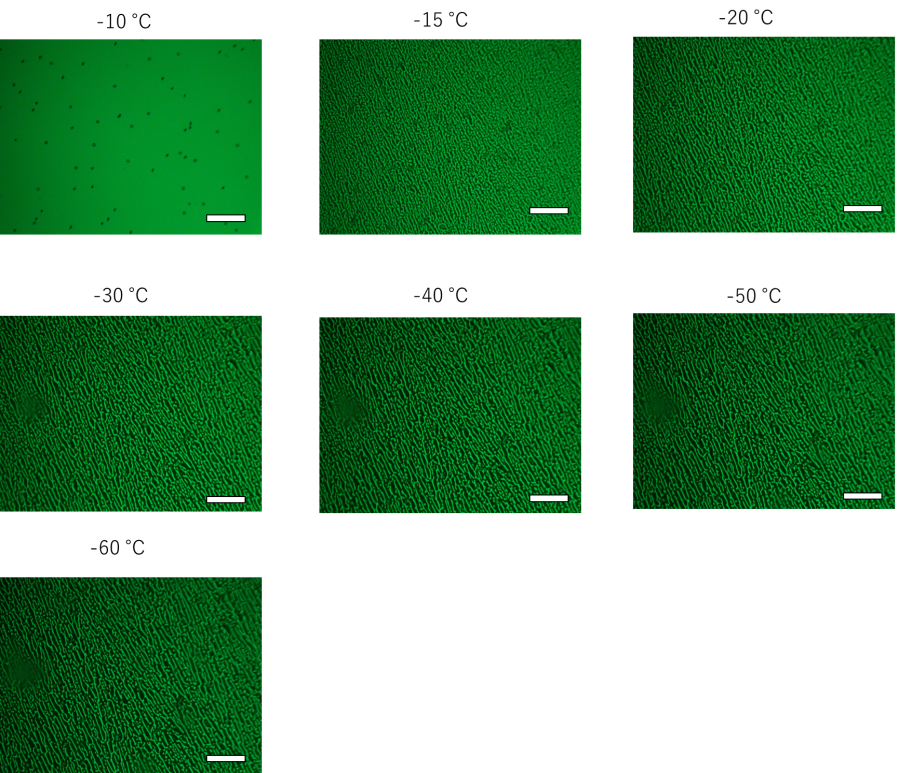


**Figure S4**. The fluorescence microscopic images of the FCS frozen at 10.0 ℃/min at every 10 ℃ during freezing processes.

10 wt.% aqueous DMSO solution containing 2 mM sodium fluorescein. Scale bar: 100 μm


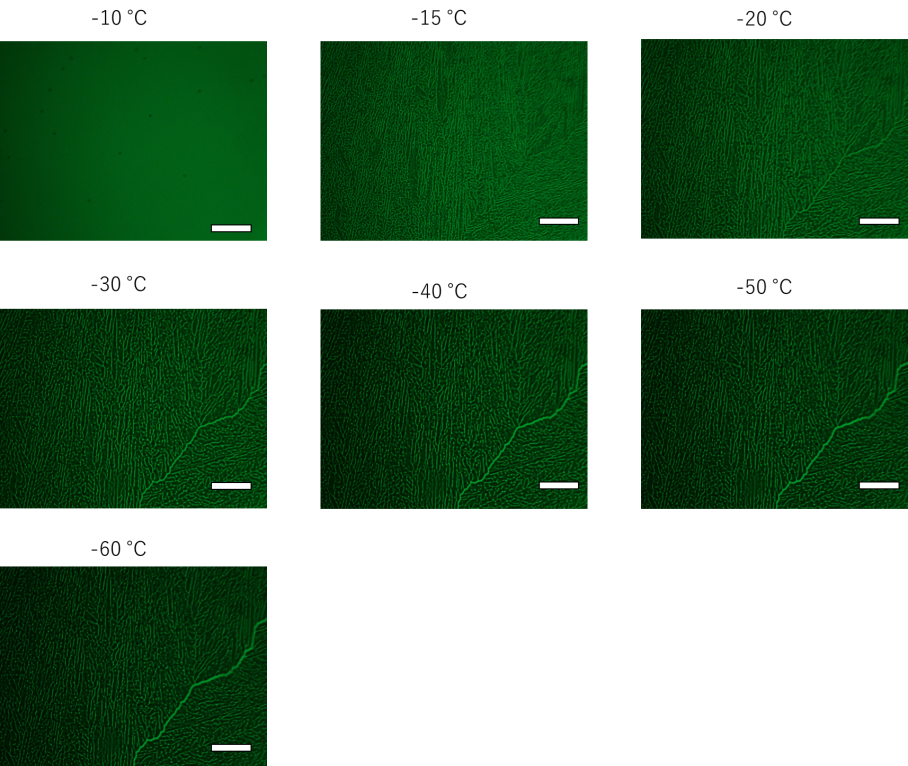


**Figure S5.** The representative morphological features of the FCS 30.0 ℃/min at every 10 ℃ during freezing processes.

10 wt.% aqueous DMSO solution containing 2 mM sodium fluorescein. Scale bar: 100 μm


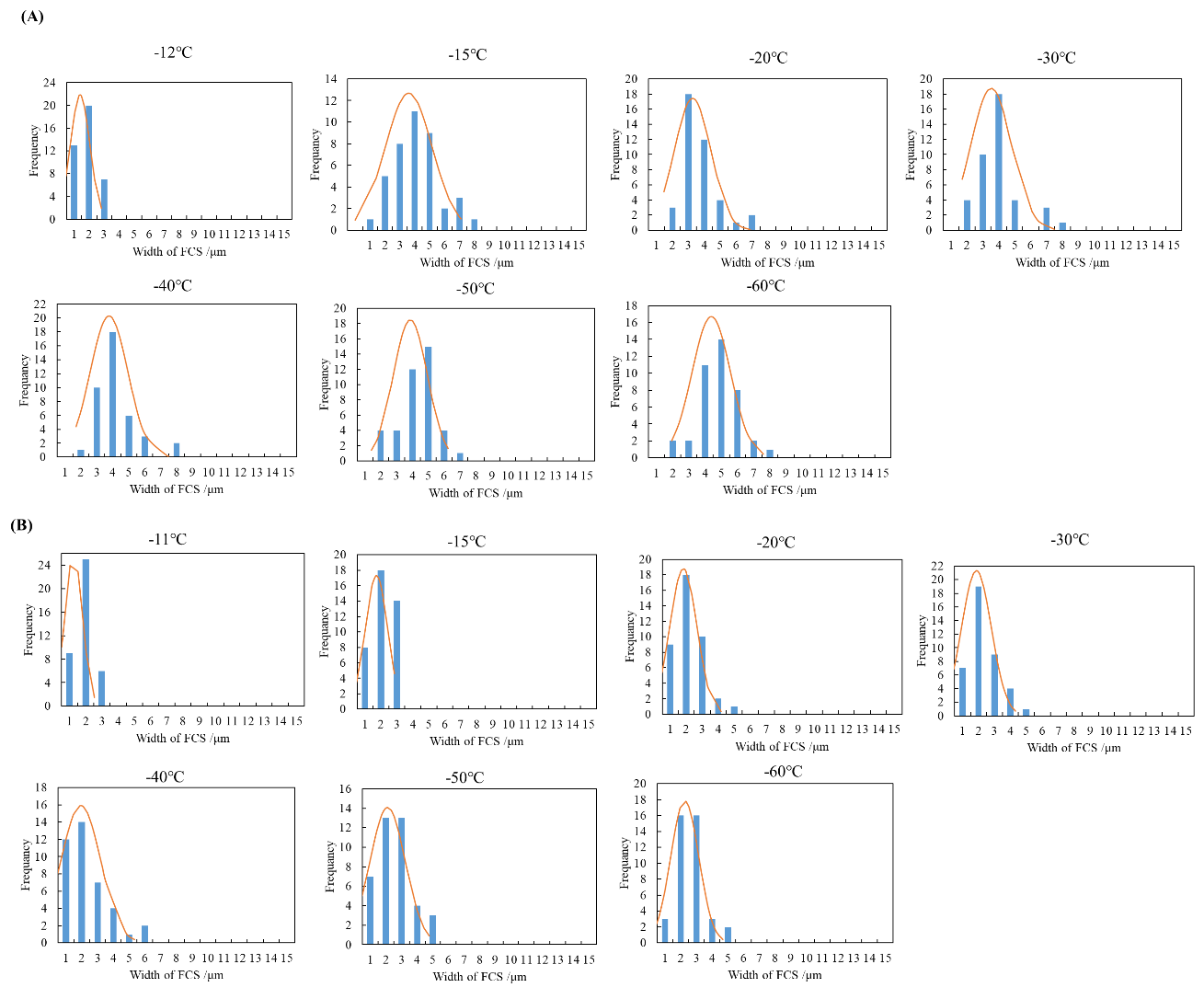


**Figure S6.** Histograms of FCS widths measured from the microscopic images at different 200 points at every 10 ℃ on freezing processes when frozen at 10.0 and 30.0 ℃/min.

(A)10.0 ℃/min (B) 30.0 ℃/min


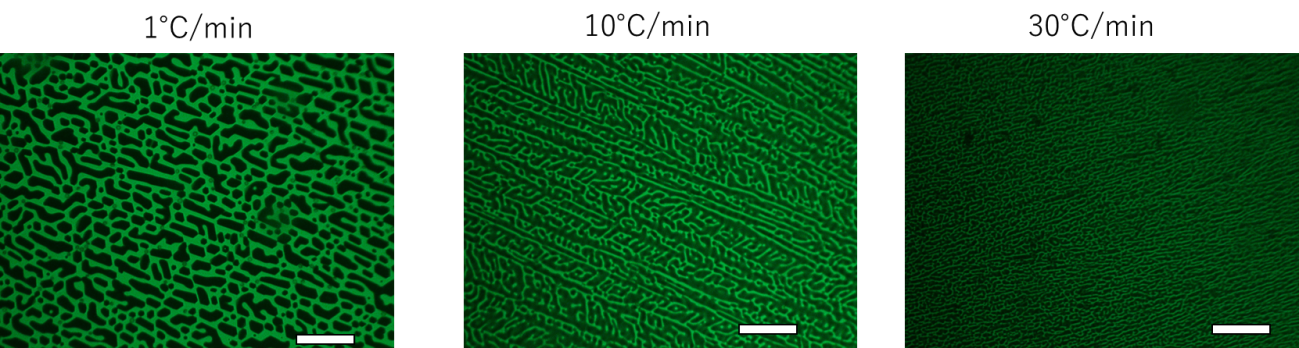


**Figure S7.** The fluorescence microscopic images of FCS when the initial concentration of DMSO was set to 20 wt.% at -60 ℃. Scale bar: 100 μm
